# Borreliae Part 1: Borrelia Lyme Group and Echidna-Reptile Group

**DOI:** 10.3390/biology10101036

**Published:** 2021-10-12

**Authors:** Giusto Trevisan, Marina Cinco, Sara Trevisini, Nicola di Meo, Karin Chersi, Maurizio Ruscio, Patrizia Forgione, Serena Bonin

**Affiliations:** 1 DSM—Department of Medical Sciences, University of Trieste, 34149 Trieste, Italy; trevisan@units.it (G.T.); ndimeo@units.it (N.d.M.); 2 DSV—Department of Life Sciences, University of Trieste, 34127 Trieste, Italy; marinacinco02@gmail.com; 3 ASUGI—Azienda Sanitaria Universitaria Giuliano Isontina, 34129 Trieste, Italy; sara.trevisini@asugi.sanita.fvg.it (S.T.); karin.chersi@asugi.sanita.fvg.it (K.C.); maurizio.ruscio@asugi.sanita.fvg.it (M.R.); 4 UOSD Dermatologia, Centro Rif. Regionale Malattia di Hansen e Lyme, P.O. dei Pellegrini, ASL Napoli 1 Centro, 80145 Naples, Italy; forgionepatrizia@virgilio.it

**Keywords:** Borrelia, Lyme, Echidna-Reptile, Spirochaeta, *Borrelia mayonii*, Baggio-Yoshinari

## Abstract

**Simple Summary:**

Borreliae are spirochaetes, which represent a heterogeneous phylum within bacteria. Spirochaetes are indeed distinguished from other bacteria for their spiral shape, which also characterizes Borreliae. This review describes briefly the organization of the phylum Spirocheteales with a digression about its pathogenicity and historical information about bacteria isolation and characterization. Among spirochaetes, Borrelia genus is here divided into three groups, namely the Lyme group (LG), the Echidna-Reptile group (REPG) and the Relapsing Fever group (RFG). Borreliae Part 1 deals with Lyme group and Echidna-Reptile group Borreliae, while the subject of Borreliae Part 2 is Relapsing Fever group and unclassified Borreliae. Lyme group Borreliae is organized here in sections describing ecology, namely tick vectors and animal hosts, epidemiology, microbiology, and Borrelia genome organization and antigen characterization. Furthermore, the main clinical manifestations in Lyme borreliosis are also described. Although included in the Lyme group due to their particular clinical features, Borrelia causing Baggio Yoshinari syndrome and *Borrelia mayonii* are described in dedicated paragraphs. The Borrelia Echidna-Reptile group has been recently characterized including spirochaetes that apparently are not pathogenic to humans, but infect reptiles and amphibians. The paragraph dedicated to this group of Borreliae describes their vectors, hosts, geographical distribution and their characteristics.

**Abstract:**

Borreliae are divided into three groups, namely the Lyme group (LG), the Echidna-Reptile group (REPG) and the Relapsing Fever group (RFG). Currently, only Borrelia of the Lyme and RF groups (not all) cause infection in humans. Borreliae of the Echidna-Reptile group represent a new monophyletic group of spirochaetes, which infect amphibians and reptiles. In addition to a general description of the phylum Spirochaetales, including a brief historical digression on spirochaetosis, in the present review Borreliae of Lyme and Echidna-Reptile groups are described, discussing the ecology with vectors and hosts as well as microbiological features and molecular characterization. Furthermore, differences between LG and RFG are discussed with respect to the clinical manifestations. In humans, LG Borreliae are organotropic and cause erythema migrans in the early phase of the disease, while RFG Borreliae give high spirochaetemia with fever, without the development of erythema migrans. With respect of LG Borreliae, recently *Borrelia mayonii*, with intermediate characteristics between LG and RFG, has been identified. As part of the LG, it gives erythema migrans but also high spirochaetemia with fever. Hard ticks are vectors for both LG and REPG groups, but in LG they are mostly *Ixodes* sp. ticks, while in REPG vectors do not belong to that genus.

## 1. Introduction

Borrelia species belong to the Spirochaetaceae family, therefore they have the characteristic spirochaete (spiral) shape. Spirochaetes cause many important diseases in humans, including syphilis and Lyme disease. Except that they contain distinctive endoflagella, no other specific molecular or biochemical characteristics are currently known for all Spirochaetes or its different families. The subdivision of the spirochetes also makes use of phylogenetic analyses based on chained sequences, and the identification of conserved signature indel (CSI), of which 38 are specific for all members of the phylum Spirochaetes, and another 16 CSI are specific for the genus Borrelia [1].

## 2. Phylum Spirochaetes

Spirochaetes constitute a heterogeneous phylum within bacteria; 16S rRNA-based sequencing is the most widely used PCR method to detect those pathogens [2]. They have only one form: the spiral cell; they have a double outer membrane different from that of the Gram negative bacteria, are anaerobic or microaerophilic, motile; they are thin and 3 to 500 μm long [3]. Spirochaetes are distinguished from other bacterial phyla by the arrangement of axial filaments (consisting of one or more fibrils), which are otherwise similar to bacterial flagella. These filaments run longitudinally along the outside of the protoplasm, but within an outer sheath, the peptidoglycan layer. Each axial fibril attaches to an opposite end and wraps around the cell body, which is enclosed by an envelope [4]. The filaments cause a twisting motion that allows the spirochaete to move by rotating in place, and pushing the bacterium forward in a corkscrew-like motion. Their number varies from 2 to more than 100 per organism, depending on the species. During reproduction, a spirochaete will undergo asexual transverse binary fission.

Spirochaetes differ in molecular characteristics including guanine-cytosine content and genome size. There are more than 200 species, many of which have not yet been characterized.

The order of Spirochaetales (Table 1) includes both aerobic and anaerobic species that are typically found in liquid environment where they are free-living (for example, mud and water) or associated with the host (blood, urine, saliva, tear fluid and lymph). Some are commensal, others are pathogens for animals and/or humans, causing diseases [5]. Spirochaetes also play important ecological roles, notably some species of Treponema (*T. saccarophilum*, *T. pectinolytic*, *T. ruminis*) live in the rumen of the cow’s stomach where they break down cellulose and other difficult-to-digest plant polysaccharides for their host [6,7,8]. There are also several saprophytic strains of Leptospirae [9].

The class of Spirochaetae currently consists of four orders and five families, as summarized in Table 1 [10].

### 2.1. Pathogenicity of Phylum Spirochaetes

Many organisms within the Spirochaetes phylum cause diseases. Pathogen (for animals and humans) members of this phylum include the following:

- Family Brachyspiraceae, *Genus Brachispira*: Spirochetosis can be associated with mild mucosal inflammation. In detail, *Brachyspira* sp. colonization was associated to mucus barrier failure. *Brachyspira aalbongi* was found in some resected appendages with possible implication in inflammation [11]. *Brachyspira pilosicoli* and *B. aalborgi* have been identified in the colonic mucosa of patients with diarrhea from irritable bowel syndrome (IBS), but not in healthy individuals [12].
- Family Brevinemataceae, *Genus Brevinema: Brevinema andersoni* can infect the short-tailed shrew (*Blarina brevicauda*) and the white-footed mouse (*Peromyscus leucopus*) [13]. Infections in humans are not known; however, *Peromyscus leucopus* is one of the main reservoirs of *Borrelia burgdorferi* s.l., which infects humans by Ixodes ticks.
- Family Leptospiraceae, *Genus Leptospira* includes both pathogenic and nonpathogenic species [14,15]. The spectrum of human diseases is extremely wide, ranging from subclinical infection to a severe syndrome of multiorgan infection with high mortality. The syndrome, icteric leptospirosis (Serogroup Icterohaemorrhagiae—*Leptospira interrogans*) can also cause renal failure [16]. It was first reported in 1886 by Adolf Weil [17]. In 1915, Inada and Ido published the first article on the discovery of the new species of Spirochaetae of Weil’s disease, which they isolated by culture [18].
- Family Spirochaetaceae: in this family, only *Treponema Genus* can be pathogenic. Among the treponematoses (diseases transmitted by *Treponema* sp.) the best known is syphilis, which is due to *Treponema pallidum*. The name of this venereal disease was given by Girolamo Fracastoro, poet and doctor from Verona, in his work “Syphilis sive Morbus Gallicus” of 1530, where the shepherd Sifilo was punished by Apollo with a disease, which rapidly spreads. From the name of that shepherd the name Syphilis was derived. The disease was likely imported from America during the travels of Cristoforo Colombo in 1492. In 1495, the disease involved the army of Charles VIII when the French king invaded Naples, and then spread rapidly throughout Europe and the world. Fritz Richard Schaudinn discovered *Spirochaeta pallida* (*Treponema pallidum*) with Paul Erich Hoffmann. Only recently it has been possible to cultivate this spirochaete [19]. There are other endemic nonvenereal treponematoses [20], as reported in Table 2.

*Treponema denticola* and other treponemes (*T. socranskii*) are responsible for buccal infections, causing acute periradicular abscesses [27] or chronic periodontitis. *Treponema denticola* is localized in the subgingival plaque and through the periplasmic flagella plays an important role in the formation of biofilm, which protects it from antibiotics [28].

### 2.2. Spirochetosis from the Clinic to Culture Isolation

Recurrent fever was a term coined by Craigie to describe the disease following an outbreak of epidemic infection in Edinburgh in the period 1843–1848, in order to distinguish it from typhus [29]. David Livingstone described fatal tick-borne fever in Angola in 1857 during his African expeditions to find the source of the Nile and to promote Christianity. In 1868, Otto Obermeier, who worked at the Berlin Charité hospital during the epidemic of the relapsing fever transmitted by lice in Berlin, highlighted at the microscope the presence of spirochaetes in the blood of sick people [30].

However, decades passed before the culture of these spirochetes in test tubes. Obermeier’s results were confirmed in 1874 by Münch and in 1876 by Motschutkoffsky, who demonstrated by microscope observation the presence of several spirochaetes in the blood of patients with relapsing fever (RF). They also demonstrated that those spirochaetes were capable of reproducing the disease by inoculating into healthy individuals the blood obtained from RF patients. Later, Ross and Milne studied RF in 1904 [31] and Dutton and Todd in 1905 [32] identified that the probable cause of the infection described by Livingstone and his team during their expedition was a spirochaete, transmitted by soft ticks of the genus *Ornithodoros*. They also showed that those ticks could also transmit the infection to monkeys. In 1906, Novy and Knappnel isolated for the first time by culture this spirochaete, called before *Protomycetum recurrentis* and then *Spirillum obermeieri* in honor of Otto Obermeier. They showed that these bacteria can grow even in absence of cells, therefore demonstrating that they were not necessarily intracellular [33]. The genus of these spirochaetes was later renamed Borrelia from the name of Amédée Borrel, who studied spirochaetes in soft argasidae ticks [34] and chickens [35], and revealed the similarity of this organism with the one described by Obermeier. Borrel documented the differences between the species of *Borrelia anserina* and the other spirochaete known at that time, *Treponema pallidum*. The name “Borrelia” was given to *Spirochaeta gallinarum* (*Borrelia gallinarum*) in 1907 by the Dutch bacteriologist Nicholaas Hendrik Swellengrebel [36]. According to Swellengrebel, indeed, *Borrelia gallinarum* did not resemble the description of the other known spirochaetes, because of its peritrichous coat, an observation made only by Borrel and not observed in other spirochaetes. Therefore, Wellengrebel was persuaded to name the genus Borrelia [37]. The problem was to keep the spirochaetes alive for a long period outside of animals and humans. Nogouchi in 1912 cultured spirochaetes in human ascitic fluid, creating subcultures maintaining their growth for many passages. The pathogenicity of these organisms is not lost in culture, although there is a tendency to attenuate the virulence if the in vitro growth lasts for a long time [38]. In the past, mainly two culture media allowed the replication of the spirochaetes and the maintenance of virulence for humans and animals [39]. In 1971 Kelly obtained an unambiguous and repeatable culture of Borrelia in an artificial medium [40], including N-acetylglucosamine as the a constituent element of peptidoglycan, which is an “ingredient” of Borrelia cell wall. Three years later, Stönner used Kelly’s medium to study the biology of *Borrelia hermsii* and noted that several hundred Borreliae were required in animal plasma to establish the spirochaetes growth in culture medium [41]. After several steps it was possible to recover “adapted” strains, suggesting that there are variants in the Borreliae population that are more suitable for living in test tubes. Kelly’s first formulation was subsequently modified by Stönner [42] and used to isolate *Borrelia burgdorferi* sensu stricto (s.s.) from *Ixodes dammini (scapularis)* ticks and then from human patients with Lyme disease [43,44]. Further modifications to Kelly’s basic composition improved the buffering capacity of *Borrelia burgdorferi*, allowing *B. burgdorferi* growth from animals [45]. This allowed isolating the recently discovered Borrelia in Barbour–Stönner–Kelly (BSK) medium [46]. Further modifications to the BSK medium followed to improve isolation [47,48].

Several additional species were subsequently described and strains of this genus are well recognized as the causative agent of Lyme borreliosis (LB) and relapsing fever (RF) in humans. In Japan (1990/1992), on Hokkaido Island, the Japanese entomologist Kenji Miyamoto isolated from *Ixodes persulcatus* ticks a new *Borrelia* sp. with characteristics different from *Borrelia burgdorferi* strains from North America, Europe and Asia [49]. In 1995, Fukunaga demonstrated that Flagellin gene from spirochaetes isolated on the Hokkaido Island differed from that of Borrelia of LB, and it was similar to that of RF Borreliae [50]. The analysis of the genome allowed for identifying it as a new species of Borrelia, which was named *Borrelia miyamotoi* [51]. *Borrelia miyamotoi* belongs to a group of spirochaetes, which cause RF, and is not transmitted by soft ticks *Ornithodoros* sp., but by hard ticks of the genus *Ixodes* sp. (hard-tick-borne relapsing fever—HTBRF), the same vector of LB. This was later cultured in different media [52,53].

## 3. Borreliaceae Family

Borreliaceae is a family of bacteria of the phylum Spirochaetales and includes two genera, *Borrelia* and *Cristispira*. Microorganisms are helical, 0.2–3 μm in diameter and 3–180 μm in length. Cells are motile, host-associated and microaerophilic, and do not have hooked ends. Periplasmic flagella overlap in the central region of the cell. Borreliae use carbohydrates and/or amino acids as sources of carbon and energy.

### 3.1. Cristispira Genus

*Cristispira* organisms (*Cristispira pectinis*) are not cultured in vitro. Genes encoding for 16S rRNA were directly amplified from bacterial DNA isolated from the oyster *Crassostrea virginica*. Sequence alignment of the abovementioned gene indicated its inclusion in the Spirochaetales order [54].

### 3.2. Borrelia Genus

**Figure 1:**
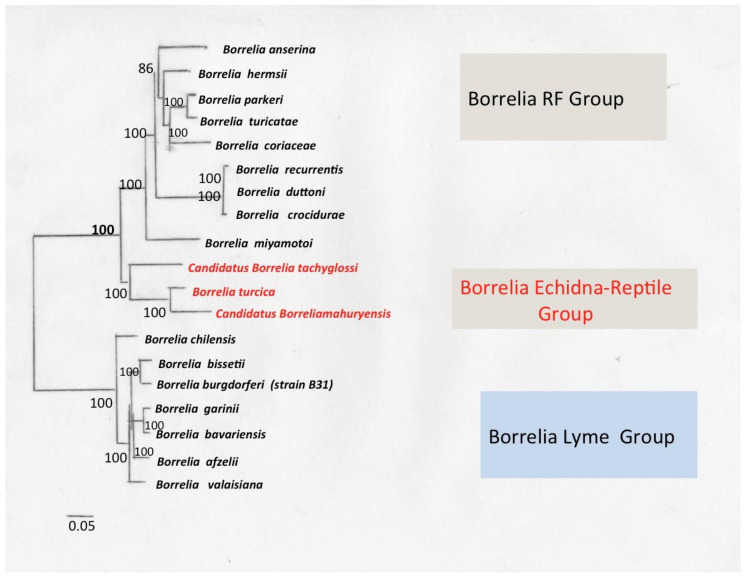
Phylogenetic relationship of 19 Borrelia genomes. The phylogenetic tree was inferred using maximum likelihood analysis of a concatenated alignment of 590 single-copy orthologous genes (197,675 AA). The number on each node represents the support of 1000 bootstrap replicates. The Echidna-Reptile group is shown in red. Elaborated from data of Binetruy et al. [56] (image by M. Cinco).

*Borrelia* genus includes Lyme (LB) and recurrent fever (RF) Borreliae, which have different clinical, biological and epidemiological characteristics. Phylogenetic data demonstrated that these two groups are genetically similar but distinct, forming independent clades that share a common ancestor (Figure 1) [55]. LB and RF Borreliae share a common set of genetic and biological characteristics that unify these organisms in a group.

All LB and RF Borrelia species have an obligate parasitic lifestyle, as they depend on their hosts for most of their nutritional needs. Borreliae are transmitted between vertebrate hosts by arthropod vectors (ticks and lice) and can be transmitted transtadially within their arthropod vectors. In nature, the vectors of LB belong to the genus *Ixodes* sp., while the vectors of RF are usually the argasid ticks (*Ornithodoros* sp.) and the human body lice *Pediculus humanus (B. recurrentis)* [57]. Borreliae also share a unique genomic structure consisting of a single highly conserved linear chromosome and several linear and circular extrachromosomal plasmids that can highly vary among strains [58]. In addition to Lyme and RF borreliosis, there are other forgotten, emerging or re-emerging borreliosis, sometimes difficult to classify as several species of Borreliae have not yet been cultured in vitro. Here, an overview of the current knowledge of borreliosis, ecology and epidemiology and their pathological potential with the main clinical aspects is made. There are Borreliae transmitted by bat ticks similar to *B. turicatae* and some of these emerging pathogens are still unnamed, such as the South African Borrelia strains found in penguins [59]. Adeolu and Gupta proposed to divide Borrelia into two genera, *Borrelia* of RF and *Borreliella* of Lyme, to reflect the genetic and phenotypic divergence between the LB and RF species [60]. However, this proposal was not followed up [61]. The methodology employed by Adeolu and Gupta specifically identifies only “conserved signature insertions/deletions (CSIs indels) and conserved signature proteins (CSPs), which are exclusive to a single Borrelia genogroup and preclude the detection of CSIs or CSPs that can be shared not exclusively between both genogroups: Lyme and RF groups. However, in the current taxonomic view it is more connotative of accurate evolutionary relationships, and the widespread genomic similarities between these two groups must be taken into account [62]. The distribution of a CSI is indicative of shared ancestry within the clade for which it is specific. In this way, the distribution of different CSIs allows for identifying different orders and families within the phylum and thus justifies the phylogenetic divisions [62].

Qin presented a more comprehensive method for delineating prokaryotic genera that measures percentage of conserved proteins (POCP) across entire pairs of genomes [63], reasoning that the degree of conservation of proteins reflects both genetic and phenotypic correlation more substantially. They also demonstrated that POCP values ≥ 50% could be considered a threshold for delimiting the prokaryotic genus, pending other genomic factors influencing POCP, such as large differences in genome size [64]. Recently, a third group of Borrelia organisms has been described which are associated with reptile hosts and echidnas (*Tachyglossus aculeatus*) and do not phylogenetically cluster within RF or LB clades [62]. *B. turcica* [65] and “*Candidatus Borrelia tachyglossi*” [66] belong to this clade together with many other genetic variants that have yet to be formally classified taxonomically [67].

Known vectors for this group include hard ticks of the genera *Amblyomma* sp., *Bothriocroton* sp. and *Hyalomma* sp.

With respect of their microbiological characteristics, Borreliae are divided into three main groups (Table 3).

There are Borrelia that have not yet been cultured, therefore it is difficult to identify the group to which they belong. Borrelia groups can be distinguished according to:

- Microbiological features;
- Vector;
- Epidemiology;
- Clinical manifestations in humans.

## 4. Lyme Group Borrelia

### 4.1. Ecology

Lyme borreliosis (LB) is a multisystem anthropozoonosis that involves the skin, joints, nervous system, heart and eyes. The clinical picture is sometimes complex and can simulate, as in syphilis, various skin and neurological diseases; consequently, LB is called the “Great Imitator”. The disease is caused by a spirochete *Borrelia burgdorferi* sensu lato (s.l.) and transmitted to humans by the bite of a hard tick of the genus *Ixodes*, which is a bloodsucking parasite of the genus *Arachnida*. LB reservoir are usually small rodents [68].

Vertebrate animals can play a double role: as ticks hosts and as spirochaetes’ hosts. There are two types of spirochaetes’ hosts as follows:

- Reservoir hosts that participate significantly in the circulation of spirochetes in nature. Ticks that feed on these animals become infected and the spirochetes multiply, disseminate in the body and persist there for a considerable period.
- Non-reservoir hosts, such as humans, where spirochetes circulation in blood is very low and ticks that feed on them do not contract spirochetosis.

Many pathogens that cause disease in humans or animals are stored in specific biological niches, from which they emerge when transmission is possible. This “reservoir function” is closely related to the association between animal species and the pathogen, which must be able to remain viable, without interfering with the survival of the host. Another essential factor is the specificity of arthropod vectors for different animal species. This is also essential for the development of any predictive model of disease risk. For a correct and complete interpretation of the epidemiology of each metazoonotic disease, the pathogen, the animal and the vector should be considered in a systematic ecological view. The ecological context, defined as the presence of microbe and animal species and their reciprocal relationships, determines the balance and interactions that influence the quantity and circulation of Borrelia, its presence in the animal basin, its spread through tick populations and the possibility of human infection [69]. Human exposure depends both on the specificity of the vector for the host and how ticks select hosts. Humans are always random hosts and the risk of exposure is based on those criteria [70]. Under these conditions, the estimated prevalence of infection based on the general characteristics of the tick population may overestimate or underestimate the risk of exposure. Theoretical models and laboratory experiments have shown that a dilution effect with decreased disease risk with increasing diversity can occur under a wide range of conditions [71]. To understand the environmental epidemiology of vector-borne diseases, it is necessary to know whether they are of low specificity or whether generalization is a phenomenon of adaptation due to the absence of the elective hosts [72]. *Borrelia burgdorferi* s.l., the agent of Lyme disease, is transmitted by *Ixodes* ticks. Interactions between *B. burgdorferi* and vectors are specific to each geographic area and determine the incidence of infections in humans [73]. *B. burgdorferi* survives in nature in a tick–mammal infection cycle. Transovarial transmission has been sporadically reported for *B. burgdorferi* s.l. [74], therefore its contribution to transmission seems to be negligible. Without transovarial transmission, the pathogen is acquired during one of the life stages of its vector at the time of engorgement on infected wild rodents or birds. The transfer of spirochetes to the vector starts after tick attachment to the host before blood meal ingestion has begun. During acquisition, spirochetes enter the tick gut from an infected host reservoir and continue to migrate until the tick is fully engorged, which usually takes 72–96 h. *B. burgdorferi* can persist in the gut throughout the life span of the arthropod, when the tick attacks other mammalian hosts, humans included, and ingests a subsequent blood meal. The spirochaetes multiply in the gut and a part of the *B. burgdorferi* reaches the salivary glands, where the spirochaete move into the new mammalian host [75].

In northeastern United States, *B. burgdorferi* is mainly maintained by a cycle involving nymphs and larvae of *Ixodes scapularis* and the white-footed mouse, *Peromyscus leucopus*. Occasionally, *I. scapularis* nymphs or adults can feed on a wide variety of vertebrates, including humans, and transmit the infection. In other parts of the United States, there are several species of the genus *Ixodes* that harbor Lyme spirochaetes, but their level of infection is low if compared to that of ticks in areas with a high incidence of human disease. Changes in tick preferences for animal species may likely explain the low prevalence of some infections. *I. pacificus* feeds on lizards (*Sceloporus occidentalis*), which are not susceptible to spirochetal infection [76]. *Ixodes neotomae*, another potential vector, feeds on rodents, but rarely bites humans [77]. In Europe and Asia, *B. burgdorferi* s.l. spirochaetes are generally maintained by *Ixodes ricinus* and *Ixodes persulcatus*, which are interspecific for different animals of the basin. LB is the most common vector-borne disease in the United States and Europe [78]. Spirochaete can spread hematologically from the tick bite site in the skin to distal tissues and organs within a host [79]. In humans, colonization of the spirochaetes in different tissues leads to multiorgan clinical manifestations, such as arthritis, carditis and neuroborreliosis [80].

In nature, ticks can acquire and transmit Lyme Borrelia between several vertebrate reservoir hosts, including avian mammal hosts and reptiles. The ability of *B. burgdorferi* to survive in ticks, to be transmitted and to systematically infect hosts is essential for the maintenance of this spirochaetes in the enzootic cycle [81]. Infection with *B. burgdorferi* s.l. is widespread in areas where Ixodes ticks and reservoirs, mainly deer mice (*Peromyscus species*) in the USA [82] and *Apodemus flavicollis* in Europe [83], are present. Ixodes ticks are essential in the transmission of LB, but they can also transmit other infectious agents in humans such as viruses (tick-borne encephalitis-TBE/FSME, Powassan) [84], Borrelia of the RF (*Borrelia miyamotoi*) [85], intracellular bacteria *(Anaplasma*/*Ehrlichia*, *Rickettsia*, *Bartonella* sp.) and Protozoa (*Babesia* sp.), which can be included in the Lyme disease coinfections [86].

### 4.2. Ticks Vector of BL Group

The main vectors transmitting BL in the United States are the black-legged ticks, *Ixodes scapularis*, in northeastern, mid-Atlantic and north-central United States, *Ixodes pacificus* on the west coast, and also in North Carolina, *Ixodes affinis* [87].

**Figure 2:**
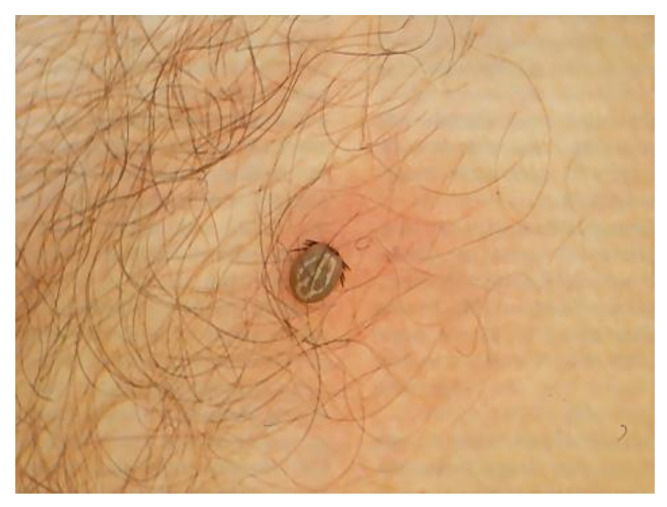
*Ixodes ricinus* on a man’s leg (photo by G. Trevisan).

In Asia and in Eastern Europe, the main vector tick is *Ixodes persulcatus* while in Western Europe and Eurasia it is *Ixodes ricinus. Dermacentor reticulatus* is the second most abundant tick species in many parts of Europe; however, its participation in transmitting BL is left open [88]. *I. ricinus* is widespread in many temperate areas of Europe and its real distribution is between the southernmost regions of Scandinavia and Finland and the most northwestern regions of Africa, and from the Atlantic coasts to the Urals. In Italy, it has been reported in almost all regions where humid forest biotopes are present, so that its frequency progressively decreases from the subalpine to the Apennine areas and from these to the southernmost areas, where it is often replaced by another species, *Ixodes gibbosus* [89]. In Italy, *Ixodes* sp. tick has been reported on humans in most Italian regions [90,91]. The wide diffusion of *I. ricinus* is due to its high ecological plasticity, being an endo-exophilic species with low parasitic specificity, infesting many different animals. *Ixodes ricinus* (Figure 2) can parasitize several mammals and occasionally humans.

### 4.3. Reservoir and Occasional Hosts

The spirochaetes of the genus Borrelia are transmitted by ticks and use reservoirs, essential for the survival of the etiological agent [92]. Borrelia infections in reservoir hosts are often asymptomatic; however, if transmitted to some aberrant hosts (for example, humans and dogs), the infection can cause various pathological syndromes. The main reservoirs are usually small rodents and birds (*Borrelia garinii* and *B. valaisiana*) [93,94]. Birds are particularly interesting as some species make long-range displacements; many have been identified as hosts of *Ixodes* sp. and may serve as reservoirs for *Borrelia* sp. [95].

In Europe, the reservoirs are mainly yellow-necked mice (*Apodemus flavicollis*), where Borrelia reproduces without causing damage to the animal and without undergoing an antibody response. Rodents, hares and some bird species have a similar role. Equally important, but controversial is the phenomenon of cofeeding; that is, the passage of the pathogen from one infected tick to another not infected when they carry out the blood meal together and nearby (transmission by cofeeding) [96]. Passerine birds and raptors are parasitized by ticks. In the Pacific Northwest region, *Ixodes auritulus* is the tick, which most frequently parasites birds of prey [97,98] and plays a vital role in maintaining the presence of Lyme disease spirochetes [99] (see Table 4).

Over 100 animal species have been identified as hosts, including rodents, birds, insectivores, carnivores and reptiles [136,137]. The bank vole (*Myodes glareolus*), natural host of *I. ricinus*, develops a natural resistance towards this tick species, resulting in the reduction of attachment success, lower tick feeding and tick survival. The yellow-necked wild mouse (*Apodemus flavicollis*), another natural host for these ticks, shows no resistance. Migratory birds can be infested with ticks, which can be infected with *Borrelia burgdorferi* s.l. These birds can act as vectors in spreading Lyme disease both as transporters for infected ticks and as reservoirs [69]. In Switzerland, 6–18% of migratory birds were found to be infested with ticks. The ticks were *Ixodes frontalis* and *Ixodes ricinus*, containing *Borrelia valaisiana*, *B. garinii* and *B. lusitaniae*. The frequent presence of *Borrelia lusitaniae* in *Ixodes ricinus* larvae suggests the possibility that migratory birds may be reservoirs of this Borrelia. Smith and colleagues examined migratory birds of the Atlantic coast of North America, finding *Ixodes uriae* and isolating *Borrelia garinii* [138].

The diversity of the vector microbiome (tick) can influence the transmission of the pathogen. *Ixodes pacificus*, the vector for Lyme disease in the Western United States, feeds on several vertebrate species that can be pathogen reservoirs, but the main blood meal host is the lizard, *Sceloporus occidentalis*, a refractory host to Borrelia Lyme group [139]. Infected ticks that feed on *Sceloporus occidentalis* eliminate *B. burgdorferi* and are no longer infectious due to complement proteins in the lizard’s innate immune system. Vector-borne pathogens are increasingly found to interact with the vector microbiome, influencing the dynamics of disease transmission [140].

### 4.4. Epidemiology

The geographic distribution of LB is related to the distribution of Ixodes vectors and to climate change. The climatic conditions limit the latitudes and altitudes in the distribution of the ticks. The wide biodiversity of the hosts and the acquisition of skills through new tick species can change the dynamics of disease transmission and this can help with understanding the dynamics and epidemiology of tick-borne diseases. For *Borrelia garinii* and *Borrelia lusitaniae*, the function of reservoir can be performed by birds, especially passerines (*Turdus merula*) [141], which can act as both reservoirs and vectors and spread Lyme disease even in very distant areas [142]. Lyme disease has a ubiquitous spread on all continents, with a particularly high incidence in the northern hemisphere, in particular North America and Europe. In North America, 90% of cases are reported from two regions in the US: the northeastern and mid-Atlantic regions and the north-central region. Both regions have expanded substantially over the past 20 years and have reached the southern parts of Canada. In Europe and Asia, the reported country-wide incidence ranges from low to negligible in the United Kingdom, Turkey, Japan, China and Mongolia, to 80 cases per 100,000 individuals in the Netherlands, Belgium, Austria, Slovenia, Italy, Lithuania and Estonia [143].

## 5. Microbiology

**Figure 3:**
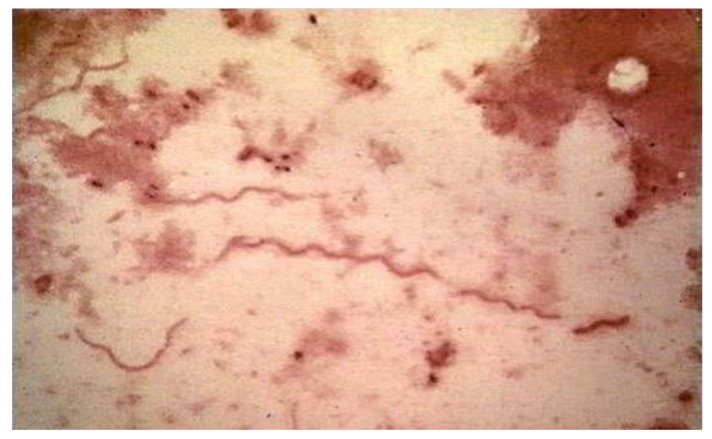
Optical microscope image of *Borrelia* sp. from *Ixodes ricinus* bowel, stained with Congo Red (photo by M. Cinco).

**Figure 4:**
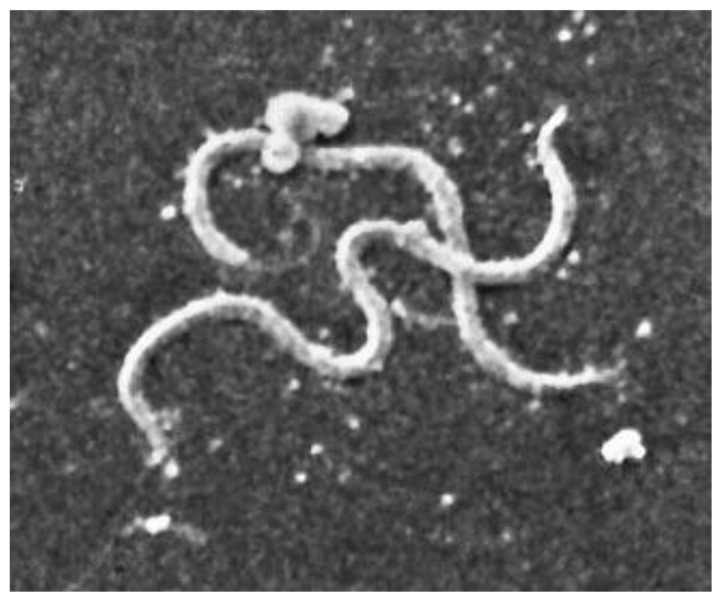
Scanning electron microscopy (SEM) image of *Borrelia burgdorferi* in BSK culture medium from the spirochaete laboratory, Trieste, Italy (photo by M. Cinco).

The infecting agent of LB is a spirochete of the order Spirochaetales, genus *Borrelia*, superspecies *Borrelia burgdorferi* s.l. (Figure 3 and Figure 4), Lyme group, a Gram-negative microorganism with a spiral shape infecting humans and animals by means of vectors (ticks).

It grows on modified Barbour–Stönner–Kelly II (BSKII) medium. Morphologically, this bacterium is similar to treponemes, from which it is distinguished by the absence of intracytoplasmic tubules and periflagellar sheath (Figure 4).

**Figure 5:**
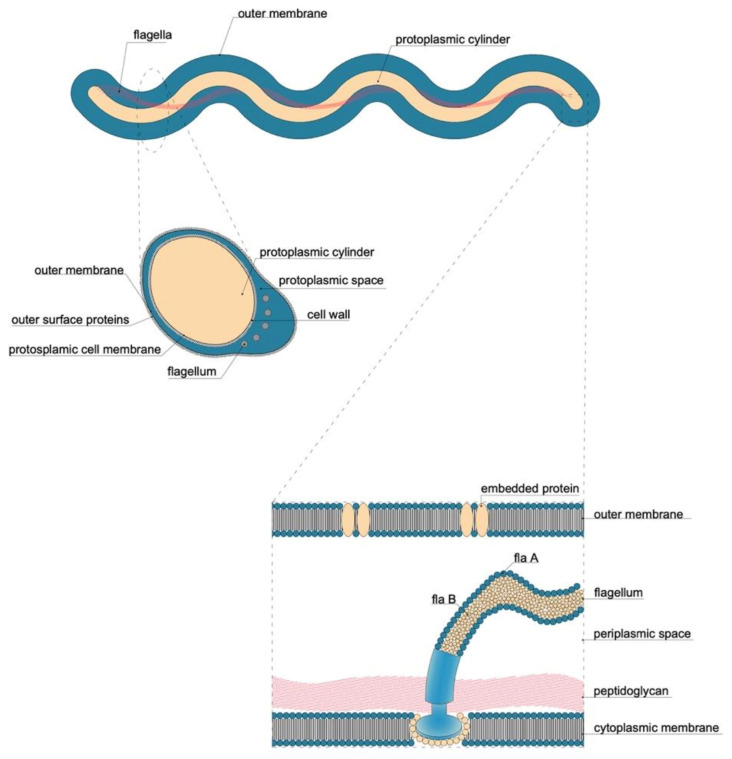
Representation of the Borrelia Lyme group structure (image by P. Forgione).

*B. burgdorferi* has an axial filament of flagella that encompasses the full length of its cell wall and outer membrane. This structure allows the spirochaete to move through viscous media, such as connective tissue. In this way, *B. burgdorferi* can spread throughout the body after a few days or weeks of infection, penetrating deeply into tissues where the immune system and antibiotics may fail to eradicate the infection. The protoplasmic cylinder is surrounded by an outer membrane similar to that of Gram negative, but free of lipopolysaccharides (LPS) and rich in lipoproteins (Osps), which are differentially expressed in the mammalian host and in the vector [144] (Figure 5).

**Figure 6:**
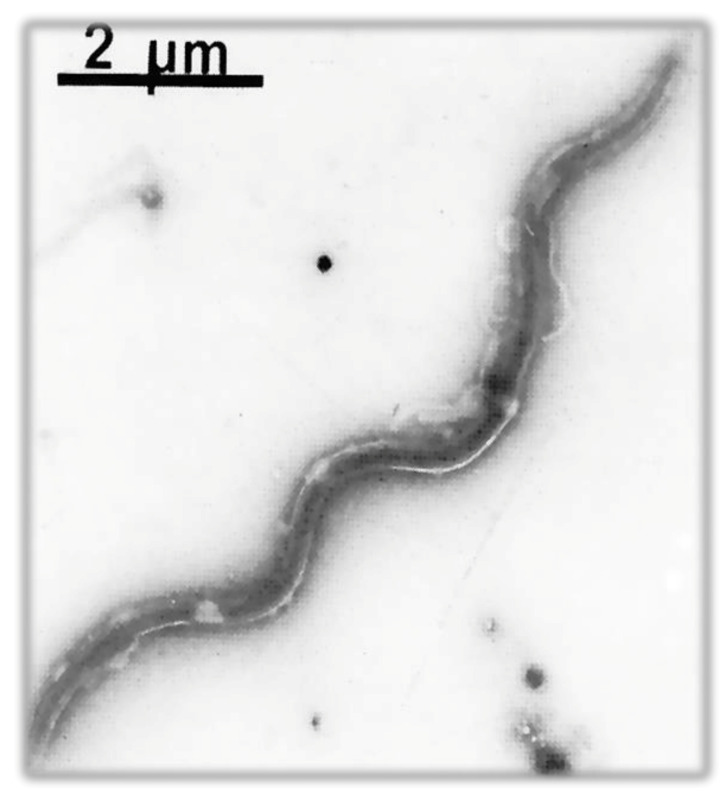
Transmission electron microscopy (TEM) image of Borrelia Lyme group, *Borrelia garinii*, BITS (Borrelia Italy Trieste strain) [146] (photo by M. Cinco).

When viewed using an electron microscope, the typical spiral shape is visible (Figure 4 and Figure 6), sharing the characteristics of spirochetes, such as the small thickness, the spiral morphology, the presence of endoflagella and the extreme lability to environmental factors; however, it is distinguished from the other spirochetes for the features described in Table 5 [145].

### 5.1. Genetic Characteristics of B. burgdorferi Sensu Lato

Over the past few decades, several virulence factors important for Lyme borreliosis have been described and studied. The investigation into the virulence traits of Lyme borreliosis expanded with the publication of Fraser’s *B. burgdorferi* genome [147] and with the subsequent complete genomic annotation [148]. *Borrelia burgdorferi* s.l. is an extremely heterogeneous bacterium; many strains have indeed been isolated and other Borrelia Lyme group species identified. The taxonomy of this microorganism is based on the genotype. By targeting the flagellin (fla) gene and the rrfA-rrlB intergenic spacer region (IGS), several genomospecies have been described [114]. The “signature” marker sequences for each genospecies are identified after sequencing the r gene [149]. Borreliae are distinguished not only by genetic characteristics, but also by the antigenic structure of the surface, the geographical distribution, the vector and the cultivability. There is also a different pathogenetic potential: *B. burgdorferi* s.s., *B. garinii* and *B. afzelii* have been shown to be responsible for pathologies for humans with different organ tropism. It has been hypothesized, following phylogenetic studies and based on the percentage of homologies between DNA sequences for the 16S subunit rRNA, that *B. burgdorferi* infection first appeared in Europe, evolving in different species and subsequently widespread in other continents [150,151].

### 5.2. Genome of Borrelia Lyme Group

Methods to detect different species of Borrrelia follow [152]:

- The intraspecific lineages of *B. burgdorferi* s.s. can be differentiated by 16S-23S ribosomal RNA spacer (IGS) and outer surface protein C gene (ospC) sequences of the plasmids.
- Multi-locus sequence typing (MLST) is used to characterize genetic variations of natural populations of a bacterial pathogen.
- Molecular phylogeny.

The Borrelia genome has a peculiar organization, unique for prokaryotes, consisting of a linear chromosome of about 910 kilobases [153] and linear as well as circular plasmids (collectively ~600 kbps), ranging from 5 to 56 kbps in size, of these several encode highly redundant sequences [147,148]. The main chromosome of the Borrelia Lyme group genospecies has low variability. This contrasts to extrachromosomal plasmids that are structurally and genetically variable and encode proteins necessary for infection of vertebrate hosts and tick vectors [154]. Genes encoding for lipoproteins of the bacterial outer membrane are located on the plasmids. One of these, the linear 49 kDa plasmid, encodes the immune-dominant surface proteins, OspA and OspB; other smaller circular plasmids seem to correlate with the virulence of *B. burgdorferi* and are lost during the in vitro cultivation. Plasmid content differs among strains and species of *Borrelia burgdorferi* s.l. However, plasmids lp54 and cp26 were observed in all the characterized strains [154,155]. Genomic comparison among species suggests that gene duplication/loss and differential expression patterns, in addition to sequence variation in conserved lipoproteins, are the primary drivers of different tissue tropisms [156]. Borrelia LG chromosome includes the 16S rRNA gene and two copies of 23S and 5S rRNA genes, which are tandemly duplicated [157]. This unique organization of the rRNA gene is the target for the molecular analysis of Borrelia. The rRNA spacer can be amplified using nested PCR, resulting in 941-bp amplicon. Restriction fragment length polymorphism (RFLP) analysis using HinfI or MseI differentiates *B. burgdorferi* s.s. strains into ribosomal spacer types, RST1 and RST2 [158]. Genotyping of Borrelia strains is important for epidemiological, clinical and evolutionary studies. Several methods are used for genotyping *B. burgdorferi* sensu lato based either on whole genome or PCR based typing [159]. A strong rate of genetic exchange, which includes the transfer of plasmids, contributes to the great pathogenicity in human and animal organisms. Long-term culture of *B. burgdorferi* results in loss of some plasmids and changes in protein expression profiles. The loss of plasmids has been associated with the inability of the microorganism to infect laboratory animals, suggesting that plasmids code for key proteins involved in virulence [160]. Nevertheless, recently a murine model to study Lyme disease has been developed to investigate the potential mechanisms of central nervous system pathologies associated with Lyme disease [161].

The most common antigens of Borrelia LG are listed hereafter according to Borrelia structures.

#### 5.2.1. Flagellum

Flagellin (p 41) is a genus-specific protein with an apparent molecular weight of 41 kDa, associated with the flagellum and located in the periplasma space. Borrelia Flagellin has some homologies with other Spirochetae (*Treponema pallidum*, *Borrelia hermsii*) inducing possible serological cross reactions. It consists of two fractions: 41a with an isoelectric point of 6.5 and the 41b with an isoelectric point of 6.6. Flagellin gene, located in the bacterial chromosome, is highly conserved, but differs in some sections among *B. burgdorferi* s.l. species. Due to its easy access, Flagellin is generally used for diagnostic purposes while its diversity can be used for identification of Borrelia species. Jaulhac et al. described a method for Borrelia genotyping by PCR fragments of the flagellin gene able of differentiating seven species of Borrelia, namely *B. garinii*, *B. afzelii*, *B. burgdorferi* s.s., *B. japonica*, *B. andersoni*, *B. valaisania* and *B. bissettii* [162]. Tryptic cleavage of the recombinant flagellin of the 41 kDa *B. burgdorferi*, expressed in *E. coli*, produced a peptide fragment that was recognized exclusively by the antisera of the Borrelia species. This peptide was designated as the 14 kDa fragment (*Borrelia burgdorferi* s.s. GeHo strain and *B. afzelii* PKo strain) [163]. The fragment is part of the variable region of flagellin, as shown by sequencing. The p14 flagellin peptide was used as antigen in ELISA and Western blot, showing a higher specificity than that obtained with intact flagellin [164].

#### 5.2.2. Extracellular Matrix Ligands (ECM)

*Borrelia burgdorferi* ligands recognizing protoglycans and glucosaminglycans (GAGs), such as Decorin and Fibronectin (fibrous proteins) have been identified and this is probably the reason why in human infection the skin and joints are affected the most [165]. Tissue invasion is facilitated by Borrelia expression of DbpA, DbpB and BBK 32 proteins which mediate the attachment to matrix proteins such as decorin and fibronectin and binding to β3 and β2 integrins, which further contribute to tissue adhesion, therefore to the persistence of the spirochete in the host [166]. P17/18 Decorin-binding protein A encoded by two operon genes is a immune-dominant antigen binding selectively to human Decorin [167]. The adhesion of pathogenic microorganisms to host cells and tissues is often mediated by the expression of surface receptors that recognize the components of the extracellular matrix. *Borrelia burgdorferi* s.s. strain B31 expresses the 47 kDa fibronectin binding protein (p47) which is localized on the outer envelope. The interaction between p47 and fibronectin is specific. The p47 peptide has been shown to match with the protein encoded by BBK32 gene of *B. burgdorferi*. The ability of the recombinant BBK32 to bind to fibronectin has been demonstrated. The protein p47, produced by the BBK32 gene, is also considered an antigen in the Western-blot serology of Lyme borreliosis (LB) [168]. The anti-Fibronectin surface protein interacts with C3b, inhibiting complement activation and accelerating its degradation.

#### 5.2.3. Outer Surface Protein (Osp)

*Borrelia burgdorferi* s.l. expresses several outer surface proteins (Osps). The distribution of the surface immunodominant proteins differs among serotypes according to the different Borrelia genospecies (*B. burgdorferi* ss, *B. garinii* and *B. afzelii*) [169,170]. It has been reported that some serotypes prevailed in a given species: OspA 1 serotype is specific for *Borrelia burgdorferi* s.l. species while the OspA 2 serotype is specific for *B. afzelii* [169]. More interestingly, specific host in nature corresponds more frequently to a given serotype. Thus, for instance, the OspA 6 serotype of *B. garinii* is found almost exclusively in the host *Ixodes ricinus*. OspC appears to play a role in vector-to-host transmission, since the protein is expressed by Borrelia only in presence of mammalian blood or tissues [171,172]. During transmission to the mammalian host, most spirochetes cease expressing OspA protein on the surface, when the nymphal tick starts blood feeding and spirochaetes in the tick’s gut begin to multiply rapidly. Simultaneous to the disappearance of OspA, the spirochaetes population in the gut of the tick begin to express OspC. Osp A, Osp B and Osp D are overexpressed in *Borrelia burgdorferi* during ticks’ colonization while Osp C, Osp E [173] and Osp F are overexpressed when *Borrelia burgdorferi* is in the mammalian host [174,175]. Osp A and Osp B are lipoproteins of the outer membrane of *B. burgdorferi*, of which the apparent molecular weight among the different species of *B. burgdorferi* sensu lato is 30–33 kDa for OspA and 34 kDa for Osp B [176]. There are two epitopes of OspA and two of OspB. Genes encoding each of them are located on a single linear plasmid of 49 Kb in a single transcriptional unit, which seems to maintain high stability even during in vitro cultivation. OspA and OspB have different molecular weight depending on the strain and species of Borrelia [177]. Although both are present in the early stages of the infection, OspA and OspB are poorly immunogenic in the initial stage, while they elicit the formation of antibodies in the late stage. OspA was used for serological diagnosis and vaccine development. Eight different OspA serotypes were identified on the basis of the differential reactivity. Among the Japanese isolates of *B. burgdorferi* s.l., OspA serotypes J1 to J11 have been recognized [178]. OspC is a surface lipoproteins complex associated with the outer membrane, of which the molecular weight varies between 21 and 25 kDa. The gene encoding OspC proteins is located on a circular single copy plasmid cp26, known to be essential for in vitro growth. OspC plays a key role in the transmission of Borrelia from tick to vertebrate and for its infectiveness in vertebrates [143]. OspC is a immunodominant antigen in the humoral IgM immune response. The expression of OspC was found to be very unstable, as they may not be expressed in culture and in the live/in vitro passage. Strains of *Borrelia burgdorferi* s.l. can be categorized based on RFLP analysis into ribosomal spacer type (RST) genotype. A correlation between RST type and invasiveness of Borrelia isolates has been demonstrated in clinical studies. RST 1 induces a greater inflammatory response than the other genotypes and patients with erythema migrans, infected with RST1 strains, had more systemic symptoms and increased levels of IFNγ as well as chemokine induced by IFNγ [179]. OspC and other external surface proteins are variable and frequently used in intraspecies population studies [158]. Genotyping can be based on the amplification and sequencing of a region of approximately 600 bp of the OspC gene. OspC typing distinguishes *B. burgdorferi* s.l. strains into 21 genetically distinct types. A correlation between OspC genotypes of *Borrelia burgdorferi* s.s. has been established: RST1 corresponds to OspC genotypes A and B; RST2 to OspC types F, H, K and N; and RST3 to the remaining 10 OspC types, including D, E, G and I [180]. OspC evokes an early response (IgM), especially in the case of erythema migrans and meningitis. The humoral response against these antigens is highly specific and qualifies them as markers of infection. The diffusion of Borrelia into the skin can be facilitated by binding with plasminogen and its activators. RST1 strains may be associated with treatment-resistant Lyme arthritis (TRLA) [158] as they stimulate an inflammatory response, resulting in the recruitment and activation of CD4 + T-cells, including some with self-reactive potential. RST1 strains (OspC type A) can play an important role, together with host factors in the symptoms of early infection and in the prevalence of TRLA. Most *B. burgdorferi* genotypes, particularly OspC type K (RST2) [180], were identified in the joint fluid of patients with Lyme arthritis, and the genotype frequencies found in joints reflected those in EM skin lesions. However, RST1 strains were most frequent in patients with antibiotic-refractory arthritis [180]. OspD (p28) is a surface lipoprotein of 28 kDa encoded by the OspD gene located on a 38 Kb linear plasmid. The protein is expressed in vitro usually after 7–9 culture passages [181]. OspD expression is generally high in *Borrelia burgdorferi* in *Ixodes scapularis*, while it is lost or reduced in human infection and in mice. Li and coworkers demonstrated that *B. burgdorferi* can compensate for the lack of OspD in both ticks and mice and that OspD may have a nonessential, secondary role in *B. burgdorferi* persistence within *I. scapularis* [175]. *Borrelia afzelii* and *B. burgdorferi* s.s. express a series of proteins, called Erps (OspE/F) and BBA68, encoded by plasmids genes. These proteins are responsible for complement resistance by binding to the complementary regulatory factor H and by means of the anticomplementary protein-like CD59, which inhibits the final assembly of the membrane attack complex (MAC) complex on the bacterial membrane. All BL group has multiple, homologous cp32 plasmids (32-kb circular plasmids), which include an Erp locus, encoding one or two surface proteins [182]. All Erp proteins are repressed during tick colonization and activated during mammal infection [183], interacting with tissue components of the mammalian host [184]. A DNA region 5′ of the start of Erp transcription, called Operator 2 and the Operator 2-binding protein, called BpaB, are essential for regulation of Erp expression [185]. OspE refers to Erp proteins with molecular weight around 20 (p19/p22) kDa. ErpP, ErpA and ErpC bind human plasminogen. Not all proteins of the OspE group have the same functions. Almost all examined OspE bind human complement factor H (CFH) via the SCR20 domain of the complement regulator [186]. They also bind CFHR-1, CFHR-2 and CFHR-5 across the C-terminal SCR domains of these proteins, but they do not bind the complement regulator FHL1. The OspE gene, located at the 5′ end of the operon, encodes a 171-amino acid protein with a theoretical mass of 19.2 kDa. The OspE p22 gene encodes a protein of 194 amino acids with an expected molecular mass of 21.8 kDa. The p22 has 98.5% homology with the *B. burgdorferi* inner membrane lipoprotein IpLA7 (p22-p22-A) with which it also shares the location in the linear chromosome of *B. burgdorferi* [187]. Anti-p22 antibodies are rarely detected in patients with erythema migrans. Seventy-five percent of patients with advanced disease tested for their antibody reactivity to the four other external surface proteins (OspA, OspB, OspE and OspF) responded to p22 or to one or more external surface proteins. Strains of *Borrelia garinii* (BITS—Trieste) include OspE genes, but have a reduced expression of OspE proteins and a reduced ability to bind FH, especially when grown in vitro for prolonged periods. Neuroinvasive strains of *B. garinii* may, however, express FH-binding proteins, which may contribute to the virulence causing neuroborreliosis [188]. OspF are surface lipoproteins of *B. burgdorferi* of 26 kDa, encoded by a polycistronic operon located in a 45 Kb plasmid. OspE and OspF genes are structurally organized in tandem as a transcriptional unit under the control of a common promoter [189].

#### 5.2.4. Heat Shock Proteins

The proteins p60 (p58) and p70 (p66) are also known as “heat-shock proteins” HSP60 and HSP70. They are two families of immunodominant proteins present throughout the *Borrelia* genus, but also in other bacterial species, such as Legionella, Listeria, Mycobacterium, Pesudomonas, Salmonella, etc. Therefore, these antigens are not specific for *B. burgdorferi* and evoke late IgG antibodies during infection. HSP 60 and HSP70 are highly conserved and are also found in the mitochondria of eukaryotes. Some associations with the onset of autoimmunity phenomena have been hypothesized for these antigens [190]. The integrin binding activity of p66 is believed to aid Borrelia escaping from the inoculation site and disseminating into tissues [191]. Five to seven HSPs were identified in *Borrelia burgdorferi* s.l.. Human immune sera collected from Lyme disease patients reacted with both 66 kDa and 60 kDa HSP, *Mycobacterium tuberculosis* and *Escherichia coli* GroEL [192].

#### 5.2.5. Other Proteins

The p39 antigen is a species-specific immunodominant protein with a molecular weight of 39 kDa, which can overlap with flagellin (41 kDa), FlaB (flagellin), Groel’s proteins (heat-shock protein—HSP). The protein p43 of *B. burgdorferi* s.l. adheres to host extracellular matrix components, including laminin, which is linked by BmpA [193]. The p83-p100 (also known as p94) is a highly specific antigen for *B. burgdorferi*, considered a “marker” of late infection [194]. It is a structural protein associated with the flagellum [195]. The use of antigens for recognizing the species of *B. burgdorferi* s.l. is not always reliable due to their variable expression of these proteins during the “in vivo–in vitro” passage and subcultivation.

#### 5.2.6. VlsE (Variable Major Protein-Like Sequence Expressed)

One of the many features of this unique pathogen is an elaborate system for antigenic variation, whereby the lipoprotein sequence bound to the VlsE surface is continuously modified through segmental gene conversion events. This constant change allows the pathogen to stay one step ahead of the acquired immune response, inducing a persistent infection. Consequently, the VlsE locus is the most evolved and diverse genetic element in the Borreliae Lyme group. VlsE p35 consists of a variable region, which changes constantly after penetration into the host, thus trying to evade the immune system; they are turned outwards and constantly change through recombinant mechanisms, following attacks by the immune system. Invariable region is masked by variable regions and protected from attack by the immune system. It is in turn composed of a mosaic of six subregions, of which IR6 (C6) is the most stable component [196].

### 5.3. Antigenic Heterogenecity of Borrelia burgdorferi

A further problem, in Europe, is the antigenic heterogenecity of *B. burgdorferi* as bands for immune dominant complexes can vary in a specific range. The OspA surface protein, for example, can vary among different species of *B. burgdorferi* s.s., *B. garinii, B. afzelii* and *B. lusitaniae* in the range of 30 to 32 kDa and the OspC from 23 to 25 kDa. This heterogeneity does not occur in North America patients, where the infection is mainly sustained by *B. burgdorferi* s.s. and the protein profile appears rather constant.

## 6. Species of Borreliae Lyme Group

Species of Borrelia LG, both pathogenic in human and not, are reported in Table 6. The haematogenic dissemination and the clinical manifestations vary between Borrelia species in human infection [99].

Hereafter, further information is given for human pathogenic Borrelia LG. *B. burgdoferi* s.s. is widespread in all continents. It has been isolated from multiple classes of vertebrate animals; therefore, it could be considered a broad spectrum species. Previous observations suggest that some *B. burgdorferi* genotypes are more prevalent in mammalian hosts such as small rodents, while others are more prevalent in avian hosts [65,192]. *Borrelia burgdorferi* s.s. has an organotropism for several organs in humans, in particular joints. The first reports of this disease, indeed, refer to some children in Connecticut in 1977 diagnosed with Lyme arthritis, appearing after erythema migrans [227,228]. Borrelia isolates associated with Lyme borreliosis have previously been divided into three genospecies based on DNA homology, namely *B. burgdorferi* s.s., *B. garinii* and VS461 group. VS461 group was identified as *Borrelia afzelii*, including 24 strains, of these six were isolated from acrodermatitis chronica atrophicans (ACA) and five from erythema migrans (EM). ACA has been frequently reported in northern Europe where *Borrelia afzelii* is often isolated, while it is rare and generally imported into the United States. *B. afzelii* was later isolated by culture in Japan from an erythema migrans. *B. afzelii* has also been isolated from *Ixodes persulcatus* ticks and small rodents, confirming that this human pathogen is retained in the rodent–tick transmission cycle [193,194]. *Borrelia bavariensis* was isolated from rodents. It is widespread in Europe and Asia and it has also been isolated both from Ixodes ticks and human specimens [198,229,230]. European *B. bavariensis* strains are highly variable in plasmid sequences in comparison to Asian isolates as a possible result of the adaptation to the tick vector. In humans, *Borrelia bavariensis* is mainly organotropic to central nervous system as is *Borrelia garinii*. *Borrelia bissettii* deriving from the DN127 group of *B. burgdorferi* s.l. was isolated from rodents in northern Colorado and recently in China from *Ixodes persulcatus* [199,200,231] and also from samples of human origin [232,233]. *Borrelia garinii* has a particular tropism in human infection for the nervous system. Events of Borrelia translocation across the blood brain barrier (BBB) involve multiple interactions between borrelial surface proteins and receptors on brain microvascular endothelial cells (hBMEC) [208,234,235]. *Borrelia lusitaniae* has been isolated in Portugal from lizards (*Podarcis muralis* and *Teira dugesii*), which can be a reservoir for this Borrelia [213,236,237]. In humans, it has been isolated in Portugal from ACA (PoHL1 strain) [238]. Phylogenetic analysis suggests *Borrelia lusitaniae* as a new species [155]. *Borrelia spielmani* frequently infects dormice, but not mice or voles. Its unique biological relationship together with genetic characterization justifies the designation of this dormouse-associated genospecies as a distinct entity. The A14S strain, later identified as *Borrelia spielmani* had already been isolated from an EM in the Netherlands in 1999, and in Hungary in 2005 [214,215,216]. *Borrelia valaisiana* was identified in Turkey from *Ixodes ricinus* tick [121] and in small rodents (*Apodemus agrarius*) in Taiwan [221,222,223]. Phylogenetic analysis showed that spirochaetes of the VS116 and M19 group from *Ixodes ricinus* in Switzerland, the Netherlands and UK were members of a distinct Borrelia species identified later as *Borrelia valaisiana* [239]. In the Netherlands, it has been detected from skin biopsy specimens from two EM patients and from one ACA patient with mixed infection (B. VS116 group and *B. afzelii*) [240]. In the Greek island of Thassos, *B. valaisiana* was identified in the cerebrospinal fluid of a patient with a slow progressive spastic paraparesis, indicating a possible association of this genospecies with disease in humans and suggesting that it might be the causative agent of neuroborreliosis [241]. Indirect evidence suggests that *B. valaisiana* is involved in some chronic clinical manifestations [242]

### 6.1. Isolated Strains of B. burgdorferi and Species Present in Italy

Isolations of *Borrelia burgdorferi* s.l. strains both from the *I. ricinus* vector (strain BITS is depicted in Figure 5), and from reservoir hosts and patients ascertained that all three main species *B. burgdorferi* s.s., *B. garinii* and *B. afzelii* are present in Italy, as shown in Table 7.

There is also indirect evidence of the presence of the VS 116 species (*B. valaisiana*). After the isolation of the first Italian tick strain called BITS, other isolations from patients followed; the samples that most likely gave positive culture were skin biopsies (EM, multiple annular/roseolar lesions, ACA). An exceptional finding was the isolation of two strains of *Borrelia burgdorferi* s.s. from myocardium, in the acute phase of the disease [240]. In this regard, the microbiological finding was the only one reporting the etiology of carditis. Currently, for classification purposes, a PCR of the sequences located in the interspace between the *rrf* and *rrl* genes was used, followed by digestion of the amplified with MseI enzyme. The electrophoretic profiles were characteristic of each species. Both molecular methods are successfully used in the identification of the infecting species of *B. burgdorferi* s.l. even if they are in small quantities in the biological sample. This is very useful as strains often do not adapt to the BSK cultural medium with the risk of being lost before their characterization.

### 6.2. Clinic

**Figure 7:**
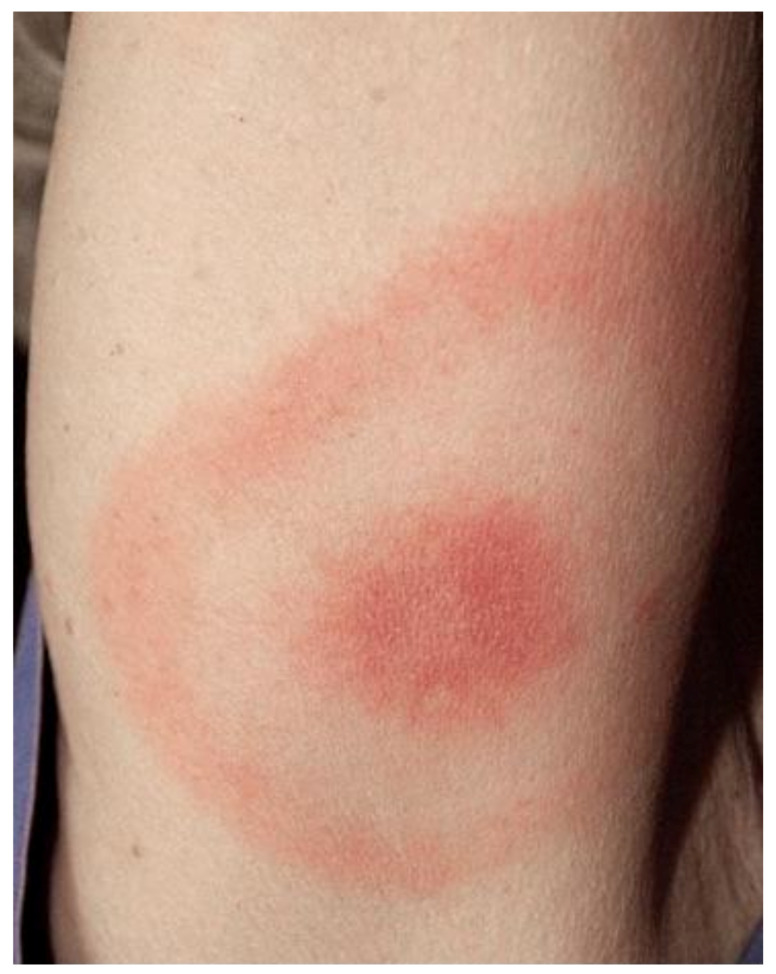
Erythema migrans of the leg (photo by G. Trevisan).

In humans, Lyme borreliosis is an infection transmitted by ticks of the genus *Ixodes*, frequently found in the northern hemisphere. Clinical features of LB are wide and variable, with clinical manifestations linked to distinct tissue tropisms of specific *Borrelia burgdorferi* s.l. genospecies [241]. The early infection is localized and, in the absence of treatment, the spirochete can spread. The organs most frequently involved are skin, joints, muscles, nervous system, heart and eyes. *B. burgdorferi* s.s. is more often associated with Lyme arthritis, *Borrelia garinii* with neuroborreliosis and *Borrelia afzelii* with ACA [242]. The clinical picture is complex and often atypical. It can mimic various diseases, especially cutaneous and neurological, therefore it is also called—analogously to syphilis—the “great imitator”. The typical first manifestation is erythema (chronicum) migrans (EM) (Figure 7), which is the most frequent and characteristic sign, but it is not always present.

**Figure 8:**
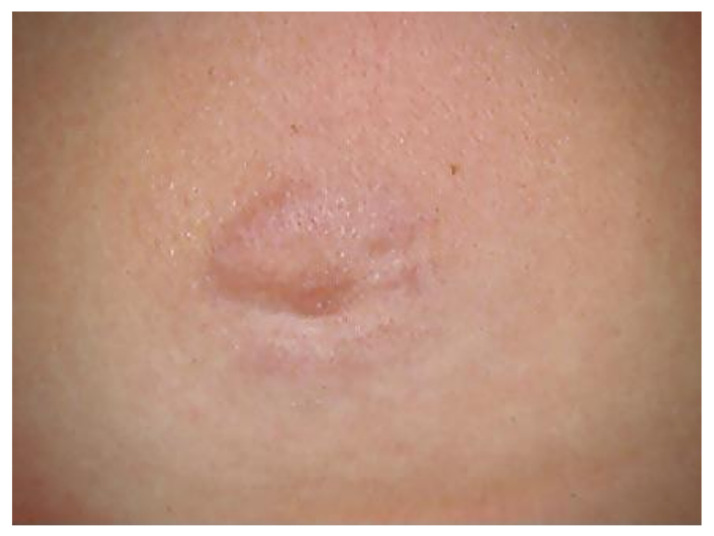
Borrelia lymphocytoma of the breast (photo by G. Trevisan).

It is a circular skin redness, which appears 5–30 days after the tick bite and tends to expand, reaching a diameter of even 40–50 cm after a few months. Although EM represents the first stage or localized early stage of Lyme borreliosis, Borrelia can also be found disseminated in blood and urine [243]. When EM is present, the diagnosis is certain and antibiotic treatment should be initiated immediately. In this phase, serological analyses are often negative. Sometimes, in the first stage it is possible to observe a follicular type of conjunctivitis. The second stage or early disseminated stage occurs approximately 3–4 weeks after infection and can last for 5–6 months, involving various organs and systems [244]. At the level of the skin, we can also have multiple annular erythema (not centered by the tick bite), and Borrelia lymphocytoma, more frequently localized in the mammary area (Figure 8); in the ear lobe, scrotal and back are observed in Eurasia [245].

**Figure 9:**
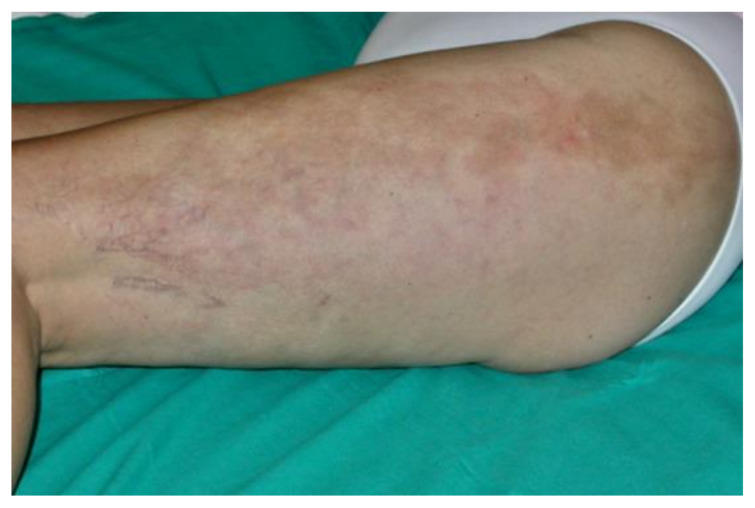
Acrodermatiitis chronica atrophicans. *Borrelia afzelii* was isolated from the biopsy specimen of the skin in BSK medium (photo by G. Trevisan).

The articular manifestations are characterized by mono- or oligoarticular, migrating myoarthralgic episodes (temporomandibular joint, wrists, elbows, shoulders, hips, knees, ankles), lasting a few days, with intervals of 2–3 weeks, which tend to shorten with the progression of the disease. The first joint affected is often the one closest to EM. Neurological manifestations (neuroborreliosis) can present with headache, paralysis of the facial nerve especially in children, and Garin–Bujadoux–Bannwarth polymeningoradiculoneuritis. In 2–10% of cases, cardiac (arrhythmias, myocarditis and pericarditis) and ocular (conjunctivitis, papillary edema, uveitis and keratitis) involvements can be observed. After 7–12 months of symptom persistence, the third stage or late stage begins. Typical skin manifestation is Pick–Herxeimer’s acrodermatitis chronica atrophicans (ACA) (Figure 9), which begins insidiously with an early inflammatory phase and infiltrated erythematous-cyanotic plaques. It occurs typically at the level of the extensor surfaces of the extremities, especially near the joints.

ACA can be uni- or bilateral. The initial lesions progressively tend to widen involving the entire acral surface, and tend to become atrophic; the skin becomes progressively smooth, thin, transparent and inelastic, and atrophy can also involve the subcutis and the underlying muscle tissue with severe limb impairment. These atrophosclerodermal forms are related to *Borrelia afzelii*, which is absent in US. The spectrum of atypical skin manifestations is wide and the correlation with marginal zone cutaneous lymphoma appears to be particularly interesting [246]. Joint manifestations in the late phase become stable, with clinical characteristics very similar to other forms of arthritis. Neurological manifestations can be different and nonspecific; in particular, psychiatric disorders such as anxiety, nonsituational panic attacks and recent cognitive disorders can be observed, and in some cases a peripheral neuropathy of the limbs, often correlated with ACA, can be observed [247].

## 7. Borrelia Lyme Group with High Spirochaetemia—*Borrelia mayonii*

This new genospecies of *B. burgdorferi* s.l. was identified in the blood of an infected patient. The same genospecies was found in ticks collected in a probable patient exposure site. In patients’ blood samples, the median copy number of spirochetes oppA1 was two orders of magnitude higher than in other Lyme borreliosis samples [219,220].

### 7.1. Clinical and Microbiological Characteristics

Borreliosis LG causes a multisystem disease characterized by tissue localization and low spirochaetemia. *Borrelia mayonii* is a new Borrelia LG, in the midwest US and causes a particular form of LB with unusually high spirochaetemia. In the Mayo Clinic from 2003 to 2014, six patients with PCR targeting the oppA1 gene produced atypical PCR results, and clinically five had fever, four diffuse or focal rashes, three had neurologic symptoms with nausea and vomiting, and two had been hospitalized for the severity of symptoms. In blood samples from patients with fever, the median number of oppA1 copies was 180 times higher in comparison to 13 samples tested positive for *Borrelia burgdorferi*. Using an eight-gene multilocus sequencing assay (MLSA), the spirochete was identified as a new *B. burgdorferi* s.l. genospecies, and referred to as *Borrelia mayonii*. This genospecies was found in ticks collected at the probable site of patient exposure [220].

### 7.2. Vectors and Reservoirs

*Ixodes scapularis* tick has been identified as the vector of *Borrelia mayonii*. The first isolation was carried out from the mouse *Peromyscus leucopus* and the American red squirrel (*Tamiasciurus hudsonicus*) from Minnesota, which could also have the function of reservoir. *Borrelia mayonii* is one of the Lyme group agents, which cause the disease in Minnesota and Wisconsin [219].

### 7.3. Genome

The higher spirochaetemia of *B. mayonii* in patients’ blood c suggests that this new genospecies must exploit strategies to overcome innate immunity, in particular complement. To elucidate the molecular mechanisms of immune evasion, various methodologies were used to phenotypically characterize *B. mayonii* and identify the determinants involved in complement interaction. *B. mayonii* resists complement-mediated killing, recruiting both key regulators of the alternative pathway (AP), factor H (FH) and FH-like protein 1 (FHL-1). The orthologous CspA protein of *B. mayonii* interacts with FH and FHL-1 by inactivating C3b, inhibiting the alternative complement pathway, but not the classical and lectin pathways. The CspA on the cell surface of *Borrelia mayonii* facilitates its serum resistance, allowing it to overcome the bactericidal activity, mediated by the complement [248].

### 7.4. Clinical Manifestations

The disease caused by *Borrelia mayonii* is characterized by fever, headache, rash and neck pain in the early stages of infection and arthritis in the later stages of infection. Unlike *B. burgdorferi* s.l., *B. mayonii* is associated with nausea and vomiting, eruptions, widespread in skin (rather than a single so-called “bull’s-eye rash”) and an increased concentration of bacteria in blood [249].

## 8. Borrelia Lyme Group—Baggio–Yoshinari Group (BYS): The Brazilian Lyme-Disease-like Illness

In Brazil, the study of LD began in 1989 and until now much of the knowledge on Brazilian borreliosis has been carried out by the team of Yoshinari. Since then, there have been several cases of human borreliosis in Brazil. The first manifestation is often EM as in classic Lyme disease; however, there are epidemiological, clinical and laboratory-related differences to define the Brazilian Lyme-like disease, which in 1993 was named Baggio–Yoshinari syndrome (BYS) [250]. In a study of 19 patients, Yoshinari reported that about 30% had skin lesions, 30% arthritis and 42% had neurological disorders; ocular symptoms were observed in 37.5% of patients, especially in the initial phase of the disease [251,252]. BYS is caused by a Borrelia which resembles *Borrelia burgdorferi* in clinical and laboratory characteristics. BYS is distinguished from LB by its prolonged clinical evolution, with a high frequency of relapses and the appearance of autoimmune manifestations. A very common symptom is headache, which can be confused with a primary chronic or analgesic overuse headache. Particular attention should be paid to patients with headache who have traveled to endemic areas. At the skin level, erythema nodosum can be observed, which is not usually observed in the classic form of LD. BYS can cause neurological, cardiac, ophthalmic, muscle and joint manifestations in humans [253].

The probable reservoir of the BYS is the capybara (*Hydrochoerus hydrochaeris*). Domestic animals can also act as vectors in the diffusion of the aforementioned spirochaete. This infection can also affect horses in Brazil, but there are currently few studies on it [254]. BYS also differs from Lyme borreliosis for the vector. This Brazilian zoonosis is not transmitted by the *Ixodes* complex, but by the *Amblyomma cajannense* tick (which also transmits the “spotted fever”) and *Rhipicephalus sanguineus*. The fact that the vector is an *Amblyomma* sp. could be one of the factors influencing some clinical aspects. BYS is widespread in Brazil and in the Amazon rainforest, but also in Colombia, Peru, Venezuela, Ecuador, Bolivia, Guyana, Suriname and French Guiana [255]. In Mexico, *Borrelia burgdorferi* s.s. has also been described in *Ambliomma cajennense*, in ticks taken from the bull (*Bos taurus*) [256]. Cases of borreliosis have also been described in Cuba, diagnosed for the clinical picture and the positivity of anti-Borrelia antibodies and transmitted by *Amblyomma cajennense*, similar to BYS [257].

### Genetic Characteristics of BYS

The Brazilian spirochetes, compared with *Borrelia burgdorferi* s.s., differ in two nucleotide bases of the flgE gene, which consists of 1119 nucleotides. This is the distinctive feature serving as a fingerprint for Brazilian Borrelia [258]. Molecular identification of *B. burgdorferi* was carried out in the blood of BYS patients by amplifying a fragment of the conserved gene that synthesizes the hooked flagellar flgE. The resulting sequences were similar to the corresponding sequences of the *B. burgdorferi* flgE gene [259]. Using antibodies against *B. burgdorferi* from North America or Europe, although relevant for the diagnosis, they show low titers and fluctuating values, which rapidly disappear in both blood and cerebrospinal fluid. Brazilian patients also have a high frequency of autoantibodies: antinuclear antibodies (ANA), anticardiolipin (ACA), cytoplasmic antineutrophils (ANCA) and antineuronal (anti-MAG). PCR methods showed a remarkable similarity between Borrelia of Baggio–Yoshinari syndrome and *Borrelia burgdorferi* s.s. However, this bacterium has not yet been cultured. The adaptation of *Borrelia burgdorferi* s.l. to different vectors and reservoirs is likely made by assuming atypical morphologies (round or cystic forms with cell wall deficiency) that show genetic adjustments [260]. These peculiarities could explain the prolonged survival of these bacteria in hosts, as well as the induction of a weak immune response and the emergence of severe reactive symptoms [261]. Borreliae can undergo structural transformations, forming dense round bodies, when found in unfavorable environments or conditions. In Brazil, the isolation of spirochetes in the form of a spiral has not yet been possible.

The concept of a new anthropozoonosis, typical of Brazilian areas, mimicking Lyme disease emerges. This is caused by Borreliae, which permanently preserve the round shape [262]. The concept of infection caused by Borrelia maintaining permanently pleomorphic forms can justify the peculiarities observed in BYS [263].

## 9. Borrelia Echidna-Reptile Group

Reptile-associated Borrelia represents a new monophyletic group of spirochaetes transmitted by several hard tick species, which has been reported to infect amphibians and reptiles in Eurasia and in the Middle East and Australia. This group (REP group) was identified after the discovery of *Borrelia turcica* in *Hyalomma aegyptium* ticks collected from turtles in Turkey [65]. This group, referred as the “Echidna-Reptile group” (REP), included initially two species: *B. turcica* and *Borrelia tachyglossi*, and some strains not yet taxonomically identified. They have been found worldwide, but not in Western Europe. It seems that this Borrelia extends also to passerine birds and to other geographical areas, such as the Americas and Africa, making it present worldwide. Borrelia species belonging to this group are not part of the Borrelia Lyme or RF groups [62], as shown even by the genome sequences [10,264]. REP group Borreliae can be differentiated from others based on the sequence and phylogenetic analysis of five genomic loci (16S rRNA, flaB, groEL, gyrB and glpQ) [67].

Vectors are hard ticks (genera *Amblyomma* sp., *Hyalomma* sp., *Bothriocroton* sp. and *Ixodes* sp.), which are mainly associated with reptiles, echidna and possibly passerine birds [66]. The description of “*Candidatus Borrelia mahuryensis*” suggests that members of this Borrelia group have a broad spectrum of hosts, which may also include a variety of passerine birds and mammals as natural hosts. There are also new strains of unidentified species of Borrelia, apparently related to this group of Borrelia and have been identified in ticks, living in Brazil [265], Argentina [266] and Mexico [267].

### 9.1. Borrelia turcica

*Borrelia turcica* is a species of reptile-associated Borrelia. The first isolation in BSKII medium was in 2003, from a hard tick, *Hyalomma aegyptium*, removed from *Testudo graeca*. Cultures were analyzed by PCR and sequencing. Tick samples were collected in northwestern Turkey, in the Istanbul area. *Borrelia turcica* is present in several southeastern European countries including Turkey, Romania, Bulgaria and Greece [268]. This new species of spirochetes (IST7T strain) was subsequently characterized in detail. Electron microscopy revealed that it is morphologically similar to other spirochetes of the genus Borrelia and has 15 to 16 flagella emerging from both polar regions. The genome comprises a linear chromosome of approximately 1 Mb; two large linear plasmids of approximately 145 and 140 kb and several small plasmids between 50 and 20 kb in size.

*Borrelia turcica* was later identified in the blood and organs of turtles exported from Jordan to Japan. However, the ecology of these spirochetes and their development in ticks or vertebrate hosts are not fully known [269]. Nymphs of *Hyalomma aegyptium* can be found not only in turtles, but also in different vertebrates and in humans. A high percentage of these ticks are infected with *Borrelia turcica*, which, however, does not appear to be pathogenic for humans. Isolates IST7 (Turkey) and 171601G (Greece) indeed showed resistance to turtle serum, but did not survive in serum from birds and humans [270].

In Argentina, ticks taken from passerine birds (Emberizidae, Turdidae, ect.) resulted to have some Borreliae. The *fla* sequences of Borrelia obtained from *Amblyomma aureolatum* confirmed a phylogenetic group with *Borrelia turcica*. The pathogenicity for humans of these Borreliae is unknown; however, adults of *Amblyomma aureolatum* tick species bite humans [266].

Borreliae with similar characteristics have also been reported in *Amblyomma maculatum* in the US and *Amblyomma longirostre* in Brazil.

### 9.2. Borrelia Tachyglossi

A new *Borrelia* sp. related to reptile-associated (REP) spirochaetes was isolated from tick *Bothriocroton concolor*, collected from Echidnas (*Tachyglossus aculeatus*) in Queensland, Australia. Echidna, also known as spiny anteaters, are egg-laying mammals classified in the order Monotremata and belonging to the Tachyglossidae family. Borrelia-specific PCR assays from these ticks confirmed the presence of a novel *Borrelia* sp. related to REP group. The three unique Borrelia 16S sequences from *Bothriocroton concolor* ticks were putatively designated *Borrelia* sp. Aus A, *Borrelia* sp. Aus B and *Borrelia* sp. Aus C, and from the *Ixodes holocyclus* tick was putatively designated *Borrelia* sp. NL230 [66].

Phylogenetic analysis of partial flaB showed that these Borreliae (Aus A–C from *B. concolor*) are a unique monophyletic clade that is closely related to the Borrelia RF and REP groups. Phylogenetic analyzes of the sequences of the flaB, groEL, gyrB and glpQ genes as well as of the linked sequences of three gene loci (16S rRNA, flaB and gyrB) confirmed that this new species of the genus *Borrelia* is closely related, but distinct from the reptile-associated (REP) and relapsing fever (RF) groups. The presence of the glpQ gene, which is absent in *Borrelia burgdorferi* s.l. spirochaetes confirmed that this new Borrelia is very dissimilar from the LB group. Based on these observations, it was called “*Candidatus Borrelia tachyglossi*”. The pathogenic potential of this bacterium is not yet known [271,272].

The role of the echidna as reservoir of this new Borrelia and the role of *Bothriocroton concolor* and *Ixodes holocyclus* as potential vectors, must be confirmed. Echidnas can also host other tick species such as *Amblyomma australiense*, *Amblyomma echidnae* and *Bothriocroton tachyglossi*. New hosts for the *Bothriocroton concolor* tick are the kangaroos (*Macropus fuliginosus*) of Kangaroo Island, South Australia [273]. This tick has a relatively wide distribution, including the coastal and subcoastal regions of Queensland and New South Wales, as well as the hinterland of New South Wales, while the distribution of *I. holocyclus* (the host of the first isolate reported by Gofton) is mainly limited to the coastal regions of eastern Australia. Therefore, several species of ticks that feed on the same host can be infected through blood meals, and the echidna serves as a vertebrate reservoir for this new Borrelia reptile group.

Another Australian Borrelia is *Borrelia queenslandica*, transmitted by the soft tick *Ornithodoros gurneyi*, identified in long-haired rats (*Rattus villosissimus*), but currently there are no precise molecular data, which would allow identification of this species [274].

### 9.3. Borrelia mahuryensis

“Candidatus *Borrelia mahuryensis*” has been isolated in culture (strain A-FGy1) from ticks associated with passerine birds in the tropical rainforests of French Guiana. This isolate is a new species of Borrelia and is closely related, although distinct, to the other two species of the REP group. *Borrelia mahuryensis* A-FGy1 genome confirmed its difference from other *Borrelia* sp., but revealed features shared with Lyme and/or RF group, and higher similarity with *B. tachyglossi* and *B. turcica* genomes, with which it shares similar gene content, including specific genes absent in Lyme or RF Borrelia groups.

*Borrelia mahuryensis* persists in French Guiana through its circulation in at least two species of ticks: *Amblyomma longirostre* and *Amblyomma geayi*, collected from passerine birds, which can be suggested as natural hosts [56]. Isolates of closely related strains of *Borrelia mahuryensis* have also recently been detected in Brazil, from *A. longirostre* ticks collected from birds [265] and in Texas from *A. maculatum* ticks [275]. Ticks *A. longirostre*, *A. geayi* and *A. maculatum* are often infected with this Borrelia, which can be transmitted transovarially to their larvae. Larvae and nymphs usually feed on passerine birds, but adults also feed on arboreal mammals, such as porcupines (for *A. longirostre* and *A. geayi*) or large mammals, such as cattle (*A. maculatum*) [276]. It is therefore possible that natural hosts of *Borrelia mahuryensis* are passerine birds, mammals or both, and that the migration of birds (*A. longirostre*, *A. geayi* and *A. maculatum*) and the transport of livestock (*A. maculatum*) can be two important factors influencing the distribution of *Borrelia mahuryensis* over long distances, thus explaining its wide geographical distribution. Borreliae REP group are widespread and biologically different. Most members of this group have been found in association with reptile and echidna hosts, but the spectrum is likely to be broader, including a variety of passerine birds and mammals as natural hosts. Infected ticks theoretically have the potential to infect animals they feed on, including humans on which *A. longirostre* and *A. maculatum* may occasionally feed; however this infection in humans is currently undocumented [277].

The presence of Borrelia REP group was also recently reported in Mexico [267,278].

## Figures and Tables

**Table 1:** Classification of bacteria of phylum Spirochaetes, class Spirochaetae.

Order	Family	Genus	Species/Groups
Brachispirales	Brachyspiraceae	*Brachispira*	*Brachispira aalborgi*
			*Brachispira pilosicoli*
Brevinematales	Brevinemataceae	*Brevinema*	*Brevinema andersoni*
Leptospirales	Leptospiraceae	*Leptonema*	*Leptonema illini*
		*Leptospira*	*Leptospira interrogans*
		*Turneriella*	*Turneriella parva*
Spirochetales	Spirochaetaceae	*Marispirochaeta*	* Marispirochaeta aestuarii *
		*Spirochaeta*	*Spirochaeta dissipatitropha*
		*Treponema*	*Treponema pallidum*
	Borreliaceae	*Cristispira*	*Cristispira pectinis*
		*Borrelia*	Lyme Group *
			Echidna-Reptile Group *
			Relapsing Fever Group

* Lyme Group Borreliae and Echidna-Reptile Group Borreliae are described in detail in this review. Relapsing Fever group Borreliae are the subject of another review.

**Table 2:** List of endemic nonvenereal treponematoses.

Disease	Treponema Species	Symptoms	Reference
Bejel (or endemic syphilis)	*Treponema pallidum* sp. *endemicum*	Mouth ulcers, mutilating nodules in bone	[21]
Yaws	*Treponema. pallidum* sp. *pertenue*	Ulcers and papilloma, mainly children	[22]
Pinta	*Treponema carateum*	Itchy patches, skin pigmentary changes	[23]
Noma (Cancrum oris)	*Treponema (Borrelia) vincentii* and *Fusobacterium necrophorum* and others	Orofacial gangrene, mainly children	[24,25,26]

**Table 3:** Borreliae classification into three main groups.

Groups	Subgroups	Humans	Clinical Aspects		Host Reservoirs	Vector Ticks	
			EM ^1^	Fever		Hard Ticks	Soft Ticks/Lice
Lyme Group	Organotropism	Yes	Yes	No	Rodents	*Ixodes* sp.	
	High Spirochaetemia	Yes	Yes	Yes	Rodents	*Ixodes* sp.	
	Baggio–Yoshinari	Yes	Yes	Yes (78%)		*Amblyomma* sp.	
Echidna-Reptile Group		Unknown			Echidna, Reptile	*Hyalomma* sp., *Bothriocroton* sp., *Amblyomma* sp.	
Relapsing Fever (RF) Group	STBRF High Spirochaetemia	Yes	No	Yes	Rodents Birds Insectivorous Ornithodoros moubata		*Ornithodoros* sp.
	HTBRF High Spirochaetemia	Yes	No	Yes	Rodents Birds Cervi (*Odocoileus virginiatus)*	*Ixodes, Amblyomma* sp.	
	Louse Fever High Spirochaetemia	Yes	No	Yes			*Pediculus* sp.
	Avian Worldwide RF High Spirochaetemia	Unknown			Birds Bats		*Argas* sp. *Carios kelleyi*

^1^ erythema migrans; RF—relapsing fever, STBRF—soft tick-borne relapsing fever; HTBRF—hard tick-borne relapsing fever.

**Table 4:** Distribution of hard ticks Borrelia LG vectors, reservoirs and Borrelia species worldwide.

Geographical Area		Hard Ticks	Reservoirs	Borrelia Species
*America*				
Canada		*Ixodes scapularis* [100], *Ixodes cookie* [98], *Ixodes spinipalpis* [101], *Ixodes angustus*, *Ixodes auritulus* [102] and *Ixodes scapularis*	*Peromyscus leucopus*, *Peromyscus maniculatus*, *Tamias striatus*, *Tamiasciurus hudsonicus* [103], *Geothlypis trichas*	*B. burgdorferi*, *B. bissettii (Lewis)*, *B. andersoni*, *B. lanei*
USA	Atlantic Coast	*Ixodes scapularis* [104]	*Peromyscus leucopus*, *Tamiasciurus hudsonicus*	*B. burgdorferi* s.s., *B. bissettii*, *B. carolinensis*, *B. kurtenbachii*, *B. mayoni*
	Northwest	*Ixodes pacificus*, *Ixodes spinipalpis* [105]	*Sciurus criseus* [106], *Sciurus carolinensis*	*B. burgdorferi*, *B. carolinensis*, *B. lanei*
	West Coast	*Ixodes pacificus*, *Ixodes spinipalpis*, *Ixodes angustus* [107]	*Peromyscus maniculatus*, *Peromyscus boylii*, *Neotoma fuscipes*, *Melospiza melodia*	*B. burgdorferi*, *B. carolinensis*, *B. lanei*
Mexico		*Ixodes kingi*, *Ixodes hearley* [108], *Ixodes scapularis* [109]	*Microtus mexicanus*, *Neotoma mexicana*, *Neotomodon alstonio*, *Peromyscus leucopus*, *Peromyscus maniculatus*, *Geothlypis trichas*	*B. burgdorferi* s.s., *B. lanei*
Brazil		*Ixodes longiscutatus* [110], *Ixodes paranaensis* [111]	*Rodents*, *Streptoprocne biscutata*	*Borrelia* sp. *Aplotipo Pampa*, *Candidatus B. ibitipoquensis*
Argentina		*Ixodes pararicinus*, *Ixodes affinis* [112]	*Turdus Birds*	*B. burgdorferi* s.l.
Uruguay		*Ixodes aragaoi* [113], *Ixodes auritulus* [114]	*Rodents*, *Passerine Birds*	*B. burgdorferi* s.l., *B. bissettii*, *B. americana*
Chile		*Ixodes stilesi* [115]	*Southern pudu deer*	*B. chilensis*
*Europe*				
Western Europe		*Ixodes ricinus* [116]	*Apodemus flavicollis*, *Turdus merula*, *Phasianus colchicus*	*B. afzelii*, *B. burgdorferi* s.s., *B. garinii*, *B. lusitasnisae*
Northern and Eastern Europe		*Ixodes ricinus*, *Ixodes persulcatus* [117]	*Myodes glareolus*	*B. garinii*, *B. afzelii*, *B. bavariensis*
Feroe Island		*Ixodes uriae (Borrelia garinii?)* [118]	*Fratercula arctica*	*B. garinii*, *B. uriae*
Russia Middle East		*Ixodes persulcatus* [119], *Ixodes pavlovskyi* [120], *Ixodes tanuki*, *Ixodes turdus*	*Myodes glareolus*, *Apodemus sylvaticus*, *Tamias sibericus*	*B. afzelii*, *B. garinii*, *B. bavariensis*, *B. tanuki*, *B. turdae*
*Asia*				
China		*Ixodes persulcatus*, *Ixodes granulatus* [121,122]	*Apodemus speciosus*, *Niviventer confucianus*, *Turdus merula*	*B. garinii*, *B. sinica*, *B. valaisiana*
Japan		*Ixodes persulcatus* [123], *Ixodes ovatus*, *Ixodes tanuki*, *Ixodes turdus* [124], *Ixodes columnae* [125]	*Apodemus speciosus*, *Apodemus ainu*	*B. garinii*, *B. tanuki*, *B. turdae*, *B. japonica*
Korea		*Ixodes nipponensis* [126,127], *Ixodes persulcatus* [128]	Wild Rodents, *Apodemus agrarius*, Migratory Birds	*B. afzelii*, *B. garinii*, *B. valaisiana*
India		*Ixodes acutitarsus*, *Ixodes kashmericus*, and *Ixodes ovatus* [129].	Rodents (Squirrels, Chipmunks)	*B. burgdorferi* s.l.
Malaysia		*Ixodes granulatus* [130].		*B. sinica*, *B. valaisiana*, *B. yangtzensis*
*Africa*				
North Africa (Tunisia Morocco)		*Ixodes ricinus* [131], *Ixodes frontalis* [132]	* Turdus merula *	*B. lusitaniae* [131], *B. burgdorferi* s.l.
South Africa		Unknown: *Ixodes rubicundus*, *Ixodes fynbosensis?* [133]	LB in horses (?) [134]	*B. burgdorferi* s.l.
*Australia*				
		Unknown: *Ixodes olocyclus*	Currently undocumented [135]	

**Table 5:** Characteristics of Borrelia Lyme bacterium.

Characteristic	Detail
Microaerophilic	
Shape	Spiral
Bacterial body length	7–24 µm
Width	0.20–0.50 µm
Propeller pitch	1.7–3.3 µm
Number of flagella	7–12
Peripheral sheath	Absent
Three-layer surface membrane	It envelops a periplasmic space, containing flagella and the protoplasmic cylinder
Cytoplasmic tubules	Absent

**Table 6:** Borrelia Lyme group A and B.

Species of Borrelia Lyme Group	Reference	Geographical Area	Human Infection	Host Reservoirs	Ticks
*Borrelia burgdorferi* sensu stricto	[197]	America, Europe, Asia, Africa	Yes	Rodents, Mammals, Birds	*I. scapularis* *I. pacificus* *I. ricinus*
*Borrelia afzelii*	[198,199]	Europe, Asia	Yes	Rodents	*I. ricinus* *I. persulcatus* *I. pavlovsky*
*Borrelia americana*	[200,201]	South Carolina, California, Poland	Unknown	Rodents, Birds	*I. pacificus*, *I. minor*
*Borrelia andersoni*	[202]	US (New York)	Unknown	Cottontail rabbits (*Sylvilagus* sp.)	*I. scapularis* *I. spinipalpis*
*Borrelia bavariensis*	[203]	Europe, Asia	Yes	Rodents, Birds	*I. ricinus* *I. persulcatus*
*Borrelia bissettii*	[204,205]	US (Colorado), Europe, China	Yes	Rodents	*I. spinipalpis*
*Borrelia californiensis*	[206,207]	US (California)	Unknown	Kangoroo Rats (*Dipodomys californicus*)	*I. jellisoni*, *I. spinipalipis* *I. pacificus*
*Borrelia carolinensis*	[208,209,210]	Southeastern region of the US, California Desert	Unknown	Rodents, Birds	*I. minor*
*Borrelia chilensis*	[211]	Chile	Unknown	Rodents, Deer	*I. stilesi*
*Borrelia finlandensis*	[212]	Finland	Unknown		*I. ricinus*
*Borrelia garinii*	[213]	Europe and Asia	Yes	Rodents, Birds	*I. ricinus* *I. persulcatus*
*Borrelia ibitipoquensis*	[111]	Brazil	Unknown	Birds (*Streptoprocne biscutata)*	*I. paranaensis*
*Borrelia japonica*	[214,215]	Japan	Unknown	Rodents (*Apodemus speciosus*, *A argenteus)*	*I. ovatus*
*Borrelia kurtembachii*	[216]	North America	Unknown	Rodents	*I. scapularis*
*Borrelia lanei*	[139,204,217]	US (California, Oregon, Washington)	Unknown		*I. spinipalpis*
*Borrelia lusitaniae*	[149,218]	South Europe, Northern Africa	Yes	Rodents, Lizards	*I. ricinus*
*Borrelia mayonii*	[219,220]	US, Europe	See Unit Lyme Borrelia Group with Spirochaetemia		
*Borrelia sinica*	[121]	China	Unknown	Rodents (*Niviventer confucianus*)	*I. granulatus* *I. ovatus*
*Borrelia spielmani*	[221,222,223]	Europe	Yes	Rodents	*I. ricinus*
*Borrelia tanukii*	[124,224,225]	Japan	Unknown	Unknown (possibly dogs and cats)	*I. tanuki*
*Borrelia turdi*	[124,225,226]	Japan, Portugal	Unknown	Migratory Birds	*I. turdus* *I. frontalis*
*Borrelia valaisiana*	[227,228,229]	Europe, Turkey, Japan	Yes	Rodents (*Apodemus agrarius)*, Song Birds	*I. ricinus* *I. granulatus*
*Borrelia yangtzensis*	[230,231,232]	China, Taiwan, Korea, Japan, Malaysia, Thailand	Unknown	Rodents, Migratory Birds	*I. granulatus* *I. nipponensis*

**Table 7:** *Borrelia burgdorferi* sensu lato strains isolated in Italy.

Year	Genus Species Strain	Isolated From	Geographic Area	References
1989	*B. garinii* BITS	*I. ricinus* Tick	Trieste	[146]
1989	*B. burgdorferi* s.s. Alcaide	Polyarthritis	Rome	[233]
1992	*B. afzelii* Nancy	Erythema migrans	Trieste	[234]
1992	*B. garinii* DA	Roseolar lesion	Trieste	[234]
1993	*B. burgdorferi* s.s. Myo I	Heart	Trieste	[235]
1993	*B. burgdorferi* s.s. Myo II	Heart	Trieste	
1993	*B. afzelii* Gualtieri	Erythema migrans	Trieste	[236]
1994	*B. garinii* Versilia	*I. ricinus*	Versilia	[237]
1997	*B. garinii*	Congenital annular erythema	Trieste	[238]
1998	*B. burgdorferi* s.s.	*Ixodes ricinus*	South Tyrol	[239]
2008	*B. afzelii*	Atrophic lesion (Anetoderma)	Trieste	[240]

## Data Availability

No new data were created or analyzed in this study. Data sharing is not applicable to this article.
